# The Need of a Medical Library in Buffalo1President’s address delivered before the eighty-fourth annual meeting of the Medical Society of the County of Erie, held at Buffalo, N. Y., Tuesday, January 10, 1905.

**Published:** 1905-03

**Authors:** William C. Krauss

**Affiliations:** Buffalo, N. Y.


### The Need of a Medical Library in Buffalo.[1]

By WILLIAM C. KRAUSS, M D., Buffalo, N. Y.

TO THE casual observer, the past decade has been characterised by a movement so overtowering that it might well be called the "era of consolidation." Whether for the public weal or woe, the idea of concentration has taken strong hold on the American mind and we have seen, especially in the commercial world the bringing together of strong industries into still stronger ones and the formation thereby of trusts and corporations of almost inconceivable power and influence.

The advantages which they derive or hope to derive are: (1) the further development of the best equipped and most available; (2) the elimination of duplication and overproduction, and (3) the saving in expense in production and disposal of their products.

But whether beneficial or detrimental to the individual, they have no doubt been the means of almost converting "agricultural America" into industrial America in the eyes of the world.

1. President's address delivered before the eighty-fourth annual meeting of the Medical Society of the County of Erie, held at Buffalo, N. Y., Tuesday, January 10, 1905.

Consolidation in the scientific and literary worlds has its advantages equally potent as in the commercial world, and out of straggling, struggling units we see the upbuilding of powerful, aggressive institutions. I know of no better illustration than in the merger of the Lennox, Astor and Tilden foundations into the great New York Public Library, destined to rank with the strongest in the world. In the literary world we have witnessed the consolidation of the *New York Medical Journal,* and the *Philadelphia Medical Journal,* the *Cleveland Journal of Medicine* and the *Cleveland Annals of Medicine.*

Medical colleges, medical societies, both local and national—have felt the warmth of union and have fused together. The movement has invaded the East as well as the South, the West as well as the North.

Medical Buffalo has been no stranger to this epidemic of consolidation and unification, and those of us who have been active during the past fifteen years have played some part in making these changes to the benefit of the profession at large. To review briefly, these events I call your attention, first, to the consolidation of the *Medical Press of Western New York* and the *Buffalo Medical and Surgical Journal* into the BUFFALO MEDICAL JOURNAL in June, 1889.

In the spring of 1892, the Buffalo Academy of Medicine was formed by the consolidation of the medical societies of Buffalo, viz.: the Buffalo Medical and Surgical Association, the Buffalo Obstetrical and Buffalo Clinical Societies. In the summer of 1898 occurred the consolidation of the medical department of the University of Buffalo, and the medical department of Niagara University, and the two medical schools have since then slept in peace and harmony.

The amalgamation of the Medical Society of the State of New York and the New York State Medical Association was decided upon at the 1904 meeting of the state society, subject to the approval of the constituent county organisation. On April 9, 1904, this society in special meeting assembled, "resolved unanimously in accepting the conditions of the aforesaid agreement between the two state organisations in so far as they applied to this society and further agreed to do its part to complete the consolidation and make it effective. At a meeting of the Erie County Medical Association, held a short time thereafter, the consolidation plan was also unanimously adopted and the association adjourned *sine die.*

That the amalgamation did not succeed eventually, was not due to any interference or lack of harmony on the part of the Erie County medical organisations.

After so much consolidation, which includes the journals, the colleges, the city societies and the county organisations, one may be pardoned for enquiring: is there anything left medically, to consolidate? Alas! there is,—there is still left one of the most important adjuncts to a medical center,—the medical library.

The library is practically a storehouse of medical literature with the advantage that all material is arranged and cared for so as to be available at a moment's notice.

The value of the medical library[1] as a factor in medical education cannot be overestimated, and the fact is too widely acknowledged to be dwelt upon at length. As Dr. Spivak[2] has pointed out, the medical school course is merely preparatory for what is to follow:

> Real knowledge of the science and art of medicine is post-graduate. . . . *Private practice* is the first institution, a teacher grim and morose, but of the highest order, if one only knows how to take advantage of its chastising lesson. *Hospital practice* is an institution wherein instruction is more systematised, the observations more certain, and the results better noted. Unfortunately there are but few in a city and less in the country . . . who can utilise it. *Medical societies* are valuable means of education, fostering and encouraging thought. But they have their drawbacks—the stated meetings that one is unable to attend; the subject that one is obliged to listen to in which he is not interested; the idle and empty discussions from which there is no escape even in the best societies, etc. There is but one grand institution that stands above all, that has all the virtues and none of the defects of those enumerated, and that is the *library*. . . . On this shelf is my physiological laboratory, on the other my biological institute; here is my anatomical theatre, there my lying-in hospital.

The records of medical progress, thousands of clinical reports, the teachings and the research of the best medical minds of all times and all places will be found in the medical library and are there available to every student.

The first medical library in the United States was founded in 1760, but it is only within the last forty years that the great medical libraries of today have been built up, and only within the last decade that the medical library movement has become active and widespread. This larger movement of recent date is still in a formative stage, and it is with the purpose of showing how, by

1. The Medical Library Movement in the United States Albert T. Huntington, Brooklyn Med. Lib. and Hist. Journal, April, 1904.

2. The Medical Libraries of the United States. Philadelphia: M. J., 1898, ii., pp. 851-858.

proper organisation and coöperation, this movement may be made more effective, that this paper is written.

Dr. Albert T. Huntington,[1] of Brooklyn, secretary of the Librarian's Association has recently compiled from various sources a list of medical libraries and their resources in number of volumes, and date of organisation. The libraries containing over 5,000 volumes are as follows:

| | Founded. | Volumes. |
|---|---|---|
| Surgeon General's office | 1863 | 145,693 |
| New York Academy of Medicine | 1847 | 80,000 |
| College of Physicians, Philadelphia | 1788 | 75,995 |
| Medical Society, Kings County, New York | 1845 | 45,000 |
| Boston Medical Library Association | 1875 | 35,000 |
| Newberry Library, Medical Department, Chicago | 1890 | 30,000 |
| Medical and Chirurgical Faculty of Maryland | 1830 | 19,761 |
| Boston Library, Medical Department | 1854 | 20,235 |
| University of Michigan, Medical Department | 1850 | 15,000 |
| Pennsylvania Hospital | 1760 | 15,500 |
| Hahnemannan Medical College, Philadelphia | 1850 | 15,000 |
| Rhode Island Medical Society | 1879 | 12,500 |
| New York State Library, Medical Department | 1891 | 12,771 |
| Cincinnati Hospital Library | 1870 | 11,881 |
| Michigan State Board of Health | 1873 | 11,740 |
| Rush Medical College Library | 1837 | 10,000 |
| Cincinnati Public Library | 1871 | 10,000 |
| University of Pennsylvania, Medical Department | 1765 | 10,000 |
| Cincinnati, Mussey Medical and Sci. Library | 1875 | 9,824 |
| Baltimore, Johns Hopkins' Hospital and Med. Society | 1893 | 9,670 |
| New York, Public Library, Medical Department | 1895 | 9,000 |
| Yale University Medical College | 1813 | 8,300 |
| Chicago, College of P. and S. Quine Library | 1895 | 8,127 |
| Worcester, Dist. M. Library | 1820 | 7,837 |
| Cleveland, M. Library Association | 1895 | 7,000 |
| Utica State Hospital Medical Library | 1843 | 6,494 |
| Syracuse University College M | 1872 | 6,321 |
| University of Buffalo, Medical Department | 1845 | 6,255 |
| Waverley, Mass., MacLean Hospital | 1880 | 6,000 |
| Massachusetts General Hospital, Treadwell Library | 1847 | 6,000 |
| Colorado Medical Library Association | 1893 | 5,342 |
| Milledgeville, Ga., State Sanitarium | 1887 | 5,256 |
| Philadelphia, Academy Nat. Sci. of Philadelphia | 1812 | 5,084 |
| Alabama State Board of Health | 1880 | 5,000 |
| Philadelphia Mercantile Library M. Department | 1821 | 5,000 |
| Pittsburg University M. Department | 1765 | 5,000 |
| Vassar Bros. Hospital, Poughkeepsie | 1897 | 5,000 |
| St. Paul, Ramsey County Medical Society | 1897 | 5,000 |
| Minneapolis, Minn., University Minn. Col. M. and S. | 1880 | 5,000 |
| Augusta, Ga., Medical College of Ga. Univ. of Ga | 1829 | 5,000 |

Dr. Huntington's summary is as follows: 215 libraries containing 1,023,295 volumes, of which 164 are medical, with 912,-330 volumes; 15 are homeopathic, with 40,653 volumes; 5 are eclectic, and the like, with 6,280 volumes; 9 are dental, with 5,093 volumes; 17 are pharmaceutical, with 54,692 volumes; 5 are veterinary, with 4,207 volumes.

1. *Medical Library and Historical Journal*, April, 1904.

If we should assume, he says, that all the libraries in the above list are in an active state of existence and their resources readily available to the profession, two striking facts are very evident. *First,* that certain centers are oversupplied with medical libraries and that the fusing of several distinct collections into one great library whose resources should be free to the whole profession could not be other than advantageous to the best interests of all concerned. *Second,* that there are certain large sections of the country which are utterly barren of adequate medical library resources.

As a matter of fact, however, probably not more than 60 per cent. of the libraries in the above list can be called active, even to a limited degree. This being the case, the need of centralisation in some localities and of extension in others becomes more apparent.

The Association of Medical Librarians, founded in 1898, held its seventh annual meeting at Atlantic City last June (1904), under the presidency of Dr. William Osler, of Baltimore. The object of the association is the "fostering of medical libraries and the maintenance of an exchange of medical literature among its members of which there are two classes: individual members or those interested in medical libraries, whether or not a medical librarian, and medical libraries, as corporate bodies, provided they contain not less than 500 volumes and have regular library hours and attendance."

The papers read at this meeting covered a wide range of subjects pertaining to medical libraries and the opinion gained ground that there was an urgent need for much aggressive spirit and work in this important field of medicine.

In his presidential address delivered at the fifty-fifth annual meeting of the American Medical Association, Dr. John H. Musser, in dwelling upon "Some Aspects of Medical Education," made reference to the Association of Medical Librarians as follows:

We are not far amiss when we call attention to an organisation which in a quiet, unobstrusive way is bringing together the members of the profession on a common ground, where tares do not spring up and thistles do not blossom, but instead in an atmosphere clear, refreshing, invigorating. Jealousies do not thrive in libraries; books soften ambitions. No stronger, more liberal friendships can develop than those that come to book lovers. We owe a debt of gratitude to the Library Association and to its distinguished president, under whose fostering care the influence and power of the association is unfolding as the hardy rose, exhaling like it a fragrance which cheers and stimulates. Of the many abundant works we owe him none, we venture, will be more satisfying to him and more lasting for good than the work of the Association of Librarians. This association should hold not its hand nor stint its heart in upholding its educational efforts.

This tribute was paid to Dr. William Osler, who was president in 1901, 1902, 1903 and under whose administration the membership increased 50 per cent. The president for 1904, was Dr. James Read Chadwick, of Boston, and it is largely to his untiring efforts that the profession of Boston owes its fine collection of medical books owned by the Boston Medical Library Association, of which he has been librarian since its founding in 1875. Among other advantages each library member is entitled, free of charge, to the following journals: *Albany Medical Annals, American Medicine, Brooklyn Medical Journal, Cleveland Journal of Medicine, Journal of the Association of Military Surgeons, Kansas City Medical Index Lancet, Medico-pharmaceutical Critic and Guide, New York and Philadelphia Medical Journal, Pennsylvania Medical Journal, St. Paul Medical Journal, University of Pennsylvania Medical Bulletin, Western Medical Review.*

In 1902 was established the *Bulletin,* devoted to the study of medical libraries—bibliography, history and biography, and one year later was merged with the *Medical Library and Historical Journal.* This journal enjoys the unique distinction of being the only magazine of its kind published in the English language. One of its objects—perhaps the most important—is to promote interest in medical libraries and their growth, the construction, administration and maintenance of libraries which have become such important adjuncts to medical education and medical progress. The list of contributors includes some of the foremost medical thinkers in America and the departments are managed in such a manner that shows it to be far from being a magazine devoted exclusively to "yesterdays."

What are other cities of the same class as Buffalo doing with this important question? Take for instance Cleveland and Cincinnati. The following letter from Dr. Dudley P. Allen, president of the Cleveland Medical Library Association is self-explanatory:

Cleveland, O., December 12, 1904.

W. C. Krauss, M. D.,

*My Dear Doctor*—I have just received your letter with reference to your address upon medical libraries. I should be glad to give you any assistance in my power since I am greatly interested in the subject. I have, however, in my possession nothing which I think would be particularly interesting or helpful to you. All I can say is that here in Cleveland we have worked along with the problem in a quiet way with one of our public libraries for a period of ten or twelve years. Seven or eight years ago we purchased property of our own upon a leading street, and now own a valuable lot with a good house upon it, which we are utilising for library purposes. We hope soon to erect a building, the basement of which shall be used as a stack for books and the upper portion of which will contain an assembly room which would probably accommodate about three hundred. This room will be used as a meeting place for our various medical organisations.

The growth of our library has increased materially since we became owners of our own property, and we now have something over 13,000 volumes.

I write you this as the basis upon which I found my judgment of the wisdom of the library having a building of its own. I think the possession of real estate gives a stability to an enterprise not to be obtained in any other way, particularly if it is well located.

Regretting that I cannot aid you more in an enterprise which always interests me, I am,

Very truly yours, D. P. Allen.

At the annual of this association, held December 10, 1904, an address was delivered by Dr. George Ben Johnston, of Richmond, Va., on the medical men contributed to the United States by the State of Virginia. This association is in the best of health.

In a paper on The Medical Library Association of Cincinnati, by Dr. Henry Wald Bettman, published in the *Lancet Clinic,* of recent date, the growth and scope of the association is portrayed.

Dr. Daniel Drake who was an ardent enthusiast in the cause of medical libraries, was active in founding the first Cincinnati Medical Library Association in 1851. On January 10, 1852, he delivered a most eloquent address on the occasion of the opening of the reading rooms of the new organisation. He especially insisted upon the duties of large cities to become the centers for the diffusion of learning, and dwelt upon the necessity for the establishment of large medical libraries from which varied learning and profounder knowledge might be gathered by the graduate in medicine.

The library of the City Hospital was started in 1870. Dr. John H. Tate was its most zealous promoter. The Cincinnati Academy of Medicine appointed a committee of six to memorialise the legislature, and an act was passed providing for the present hospital library. In 1874 the library contained 1,511 volumes; in 1884, 4,320 volumes; in 1894, 9,316 volumes; and at present nearly 15,000 bound volumes. Dr. William Carson guided the destinies of the library with great ability and foresight from 1880 to the time of his death, in 1893. Dr. P. S. Conner has been librarian since that time. The library has grown steadily in usefulness. It contains comparatively little "dead material." It possesses complete files of most of the practical journals in medicine and surgery in German, French and English.

The present Medical Library Association was organised in May, 1893, in the hope of enlarging the usefulness of the existing medical libraries. Its functions are: (1) to render the existing collections as accessible as possible by the preparation of the necessary indexes and catalogues; (2) to provide an attendant, trained in library methods, to assist those desiring to look up special subjects; (3) to extend the present collections by subscribing for journals not now taken; (4) to form a permanent organisation for the reception of gifts of books, or of endowments, and to collect medical photographs and instruments of historic interest.

An ultimate aim of the association would be to unite all local medical collections under one roof, in a fire-proof building, centrally located.

---

What a few other cities are doing: in Montreal, the McGill Journal Club has lately been established for the purpose of augmenting the journal resources of the Library of the medical department of McGill University. The club starts with a membership of about 60 and with annual dues of $2.00. The funds are to be devoted to the subscription to standard journals in the various branches of medicine not already taken by the library. When the resources of a library are limited, such organisations as this form a very valuable auxiliary. In Baltimore, the Book and Journal Club for many years has been of invaluable service to the Library of the Medical and Chirurgical Faculty of Maryland. Two years ago the Medical Library Association of Brooklyn was founded to perform a like service for the Library of the Medical Society of the County of Kings.

The last annual report of the committee on library of the Rhode Island Medical Society, published in the *Providence Medical Journal* (1904, v., pp. 116-118), shows a satisfactory growth of the library. Notable among the additions of books during the year was a collection of works on sanitary science, donated by Dr. Charles V. Chapin. One hundred and seventy-five periodicals are on current file. Dr. Horace G. Miller was re-elected chairman of the committee.

According to the *St. Paul Medical Journal* (1904, vi., pp. 206-207), the library of the Ramsey County Medical Society now contains upwards of 4,500 volumes and receives about 125 medical journals regularly. A permanent home for the library is already assured. The society has established a building fund and, when sufficient amount of money has been secured, intends to buy a piece of ground and erect a building.

Other medical libraries, with an income inadequate to provide the large number of special journals necessary to every well-equipped working library, will profit by adopting the same measures. It means a number of public-spirited physicians who are willing to contribute more than their share for the benefit of all.

Library associations are being organised in many of the southern cities, notably, Atlanta and Birmingham, Ala.

What is Buffalo doing with this movement which has taken such firm hold on the profession and which bids fair to increase in the next decade to a greater degree even than in the last? To this question we can only reply that as yet we are only watching the movement and have not as yet taken our place in the procession among the leaders where we justly belong. I have the honor to present to you an inventory of our library facilities, kindly prepared by Miss Emma L. Chappell, of the University library and Mr. E. P. Van Duzee, of the Grosvenor library.

At the present time there are in Buffalo existing under separate management the library of the medical department of the University of Buffalo, the medical library of the Grosvenor reference library, the medical library of the Buffalo Library Association, the library of the Erie County Medical Society, and the library of the Buffalo Academy of Medicine.

This list does not include the libraries of the Buffalo State Hospital for the Insane, numbering 1,000 volumes, nor the library of the German Hospital, numbering 400 volumes, nor that of the Buffalo General Hospital, numbering—volumes, nor the State Cancer Laboratory, containing 250 volumes.

Medical library, University of Buffalo, number of volumes, January 1, 1905, 6,896. Number of files complete of (1) American journals, 11; (2) foreign journals, 12. Number of files incomplete, (1) American journals, 122; (2) foreign, 32. Number of additions annually to library, 279. Amount appropriated for purchase of medical books, state, $100.00; medical department, $200.00; total, $300.00. Number of journals subscribed for (1) American, 43; (2) foreign, 32; contributions: Dr. Geo. N. Burwell, interest on $2,500.00 and medical library containing 719 volumes.

Grosvenor Library—medical department.—Total number of volumes, 6,432; total number of pamphlets, about 1,200; complete files of American periodicals, 14; complete files of foreign periodicals (*Lancet*), 1; incomplete files of American periodicals, 265; incomplete files of foreign periodicals, 52; number of additions, manuals and other books not periodicals, 150; amount expended annually for books not periodicals, $300.00 to $400.00; principal donors: Dr. Theo. G. Lewis, in addition to his original gift of about 700 volumes of dental books he has kept up the sets of periodicals and secured for the library the new dental books as they have appeared. The New York Academy of Medicine has given the library about 1,000 volumes, mostly periodicals; Mrs. F. W. Abbott, about 200 volumes; Mrs. H. A. Foster, about 100 volumes; Dr. H. D. Ingraham, Mrs. A. R. Wright, about 150 volumes; Dr. Roswell Park, 125 or more volumes; Dr. Alvin A. Hubbell, 100 or more volumes; Dr. Benedict, Dr. Carpenter, Dr. King, Dr. William C. Krauss, and Dr. Lucien Howe and many others have given to the library valuable additions in books and periodicals. Special mention should be made of Dr. William Warren Potter, who keeps the library supplied with many files of periodicals regularly, besides contributing many books and reports of much value.

The library of the Erie County Medical Society is housed temporarily at the university library and consists of about 800 volumes.

The library of the Academy of Medicine, donated by Dr. F. W. Bartlett, is housed temporarily at the Grosvenor Library and consists of 186 bound volumes and 1,063 unbound periodicals and pamphlets.

Among the private libraries of large proportion in Buffalo may be mentioned those of Dr. William Warren Potter on general medicine and gynecology; Dr. Roswell Park on surgery; Dr. Lucien Howe on ophthalmology; Dr. Alvin A. Hubbell on ophthalmology; Dr. Charles G. Stockton on general medicine; Dr. Ernest Wende on dermatology and microscopy; Dr. M. D. Mann on gynecology.

The questions pertinent to the hour, it seems to me, are: (1) shall Buffalo have a medical library worthy of its importance as a medical center? (2) shall this library be accomplished through consolidation of the existing libraries? (3) what library offers enough advantages over the others to be selected as the depository for the books? (4) shall a medical library association be formed in Buffalo, patterned after those so successful in other cities? (5) is a medical home necessary or desirable to house such a library in case no existing library is acceptable?

Shall Buffalo have a medical library worthy of its importance as a medical center?

This question will scarcely call for debate. There are always two things which challenge admiration in a medical center by the profession as a whole: first, the medical home, and second, the medical library.

Take for instance the New York Academy of Medicine, housed in its own beautiful building, with a library containing over 80,000 bound volumes, about 36,000 pamphlets, and which subscribes regularly to more than 1,200 current periodicals, comprising nearly all the medical journals, transactions of medical societies, hospital and boards of health reports published throughout the world.

The medical investigator in New York has little need to call to his assistance the resources of the surgeon-general's library of Washington. New York physicians may justly feel proud, as they indeed do, and their efforts should be emulated by the physicians of other cities.

Shall this library be accomplished through the consolidation of the existing libraries?

If practicable this would be the ideal plan. It would result in a library of about 15,000 volumes. It would mean the expenditure of $700.00 for new books and periodicals. It would mean the filling out of incomplete files of American and foreign periodicals. It would avoid duplication in books and periodicals. It would mean a considerable saving in cost of management. It would save time and trouble to the physician in looking up his references.

These books need not be given outright, but held in trust by the receiving library, or they may be sold as was done by the physicians of the German Hospital and Dispensary, of New York, who after striving for over thirty years to collect valuable medical books, decided that it was best to dispose of the library. It was accordingly sold to the King's County Medical Society for $3,000, and this sum was then presented to the New York Academy of Medicine, with the understanding that it should be known as the German Hospital and Dispensary Library Fund.

What library possesses advantages sufficient to be selected as the depository of the books?

But two libraries can be considered. The library of the University of Buffalo is not a public library in the true sense of the word. Its hours are restricted, and its accommodations are limited. The attendants, Miss Chappell and Miss Staffeldt, are most courteous and obliging. It possesses one great advantage over the Grosvenor, in that books and periodicals may be taken from the building.

The Grosvenor Library is located centrally and is therefore very accessible. The library is open from 9 a. m. to 10 p. m. during the week and from 2 to 6 p. m. on Sundays. It is well lighted and evenly heated. The attendants are equally courteous and obliging. The facilities of the library are greater, the resources larger and the growth more vigorous than the university library. It has the backing of the city government and the moral support of the whole profession.

In order to make it more useful, especially to those who wish to undertake the investigation of any subject, as in the preparation of a paper, it has sent out cards, giving name, address, and the subject on which material is desired, and the library attendants will look up the matter in advance, so the resources of the library on this subject will be at the disposal of the physician at the time named.

Is the organisation of a library association desirable?

If we desire to make progress along the lines indicated, such an association is not only desirable but necessary. If these associations are successful in Brooklyn, Baltimore, Cleveland, Cincinnati and other cities, why not in Buffalo?

Is a medical home necessary or desirable to house such a library, in case no existing library is acceptable?

There are two requisites for the establishment and permanent success of a medical library: first, a desire on the part of the local profession to have a library; second, the control of that library, wherever the books are housed, by the medical profession. Browning, in an admirable paper on the subject, contends "that experience so far shows the independent medical library to offer the most promise of permanency and usefulness. There can be no question but that, whenever practicable, this particular form of special library will be more successful and permanent if conducted as a separate library and under the auspices of some general medical organisation." What can be done by a county medical society is evidenced by the Medical Society of the County of Kings (700 members) which owns its own building, valued at $100,000, in which is housed one of the leading medical libraries of the world.

At the 1904 meeting of the Association of Medical Librarians this subject was warmly debated by Canadians and Americans, and the consensus of opinion was that wherever the medical library was housed, or however it was supported, the time would eventually come when it would become necessary for the medical profession itself to take care of the medical library in order to ensure its lasting success.

I am happy to state that the Buffalo Academy of Medicine has a building fund amounting to $2,700.00 available for this purpose. In time possibly the solution of this whole question may be accomplished in this way.

If no consolidation is feasible, and the organisation of a library association not advisable at this time, then a sort of community of interest plan might be followed, such as a (1) card index catalogue of not only the public libraries, but of the private libraries as well, this to include especially the foreign periodicals, copies of the old classics, first editions of the old masters, and the early American contributions to medicine; this catalogue to be not only a subject-card catalogue, but author catalogue as well, and each library, *i. e.,* the University and Grosvenor to be supplied with one; (2) some arrangement whereby the periodicals subscribed for, especially the foreign, will not be duplicated thus extending the list of periodicals to nearly double of what it is at the present time; (3) the development of each library along certain lines adapted to the wants of those frequenting them; for instance, the University Library in the textbook line, the Grosvenor in reference bibliography and biography; (4) acquainting the profession with a better understanding of the use of the Index Medicus and the Index Catalogue of the library of the surgeon-general's office; the librarian of the Grosvenor Library will be pleased at any time to send for books, the physician paying the actual express charges only to and from Washington; (5) to preserve, as far as possible, the literary efforts of the Buffalo and Erie county profession, all publications should be deposited either in the Grosvenor or both libraries by the respective authors. Reprints of all publications by the local profession should be sent to the Grosvenor, where they will be bound and stored, a separate section being devoted to these volumes.

479 Delaware Avenue.

---

The hypodermic injection of brandy, whiskey or sulphuric ether is one of the best methods of combating intense shock and collapse, but the surgeon should always remember to inject them deep into the muscles, as they may cause sloughing of the skin if injected beneath it, and they should not be introduced in the neighborhood of any important nerve, as they have been known to cause paralysis or neuritis.—*Jour. Medicine and Science.*

